# The complete mitochondrial genome of *Panax ginseng* (Apiales, Araliaceae): an important medicinal plant

**DOI:** 10.1080/23802359.2021.1981167

**Published:** 2021-09-27

**Authors:** Woojong Jang, Hyun Oh Lee, Jung-Woo Lee, Nayeong Kwon, Dong-Hwi Kim, Kyong-Hwan Bang, Ick-Hyun Jo

**Affiliations:** a Department of Herbal Crop Research, National Institute of Horticultural and Herbal Science (NIHHS), Rural Development Administration (RDA), Eumseong, Korea; b Phyzen Genomics Institute, Seongnam, Korea

**Keywords:** Ginseng, mitochondrial genome, medicinal plant, *Panax ginseng*, phylogenetic analysis

## Abstract

Ginseng (*Panax ginseng* C. A. Meyer) is a multifunctional medicinal herb used worldwide and is an economically important high-value crop in Korea. Here, we presented the mitochondrial genome of *P. ginseng* landrace ‘Jakyung’, which is one of the most common cultivars cultivated in Korean farms. The complete mitochondrial genome sequence was 464,661 bp in length and had a single circular form. The ginseng mitochondrial genome encoded 72 unique genes, including 45 protein-coding genes, 24 tRNA genes, and three rRNA genes. Nucleotide composition analysis revealed a GC content of 45.1%, with a slightly higher A + T bias (A, 27.1%; T, 27.8%; G, 22.5%; C, 22.6%). Phylogenetic analysis showed that *P. ginseng* was closely related to *Daucus carota* in the Apiales. This complete mitochondrial genome sequence of *P. ginseng* provides valuable genetic information for further studies of this important medicinal plant.

*Panax ginseng* C. A. Meyer, which belongs to the Araliaceae family, is a well-known medicinal herb used worldwide. This slow-growing and shade-requiring perennial plant is mainly distributed in Northeast Asia (Yun 2001). Ginseng has various pharmacological effects on humans (Shin et al. 2000; Yuan et al. 2012) and has a high value in the market (Baeg and So 2013). In Korea, ginseng has been cultivated for hundreds of years as a major medicinal crop, occupying a substantial portion of the agricultural economy. Although diverse ginseng cultivars have been developed, the landrace ‘Jakyung’ is still the most common cultivar cultivated at local farms (Zhang et al. 2020). ‘Jakyung’ has a purple stem and bears red mature fruits. Until now, many studies have been focused on pharmacological effects of ginseng, but genetic researches are still limited and lacking. Here, we characterized the mitochondrial genome sequence of the *P. ginseng* landrace ‘Jakyung’ and analyzed the phylogenetic relationships among related species.

A specimen of *P. ginseng* landrace ‘Jakyung’ was collected from the National Institute of Horticultural and Herbal Science research field in Korea (Eumseong, 127°45′13″E, 36°56′36″N) and was deposited at the Ginseng Research Division in the Department of Herbal Crop Research under voucher number MPS002301 (https://www.nihhs.go.kr/eng/about/nihhsLocation.do, Ick-Hyun Jo, intron@korea.kr). DNA extraction was performed from frozen leaves using a DNeasy Plant Mini Kit (Qiagen, Hilden, Germany) following the manufacturer’s instructions. Oxford Nanopore and Illumina MiSeq libraries were prepared using the Rapid Sequencing Kit (SQK-RAD004, Oxford Nanopore Technologies (ONT), Oxford, UK) and the TruSeq Nano DNA Kit (Illumina, San Diego, CA, USA), respectively, following the manufacturer’s instructions. Raw sequencing data was generated using Oxford Nanopore MinION and Illumina MiSeq platforms. The assembly process was carried out based on a hybrid method using ONT long-reads and Illumina short-reads. Raw ONT long-reads were trimmed using Porechop version 0.2.3 (https://github.com/rrwick/Porechop) with default parameters. The trimmed ONT reads were assembled using NextDenovo version 2.3.1 (https://github.com/Nextomics/NextDenovo), and mitochondrial-derived contigs were selected by BLASTN searches (Altschul et al. 1990) against the National Center for Biotechnology Information (NCBI) mitochondrion database (https://ftp.ncbi.nlm.nih.gov/refseq/release/mitochondrion/) with an E-value threshold of 1E-6. A draft mitochondrial genome was assembled through a manual joining process using overlapped contigs. Raw Illumina short-reads were trimmed and mapped on the draft mitochondrial genome using quality_trim and clc_ref_assemble tools with default parameters, respectively, in the CLC Assembly Cell package version 4.2.1 (CLC Inc., Aarhus, Denmark) for manual error correction. The complete mitochondrial genome sequence was annotated by using GeSeq with default parameters (Tillich et al. 2017). Phylogenetic analysis was performed by using the maximum likelihood method with 1000 bootstrap replicates through MEGA7 (Kumar et al. 2016). Eighteen orthologous protein-coding genes in mitochondrial genomes was extracted from 14 species and aligned each other for comparative analysis. Two species belonging to the Campanulaceae family were used as outgroup.

The complete mitochondrial genome sequence of *P. ginseng* landrace ‘Jakyung’ was deposited in the NCBI database under accession number MZ389476. The mitochondrial genome was 464,661 bp long and had a single circular form, which is common in general organisms. The ginseng mitochondrial genome contained 72 unique genes encoding 45 proteins, 24 tRNAs, and three rRNAs. Among these, eight genes (*cob*, *rpl10*, *rrn26*, *trnE*-UUC, *trnM*-CAU, *trnP*-UGG, *trnQ*-UUG, and *trnY*-GUA) were duplicated, and seven copies of the *trnM*-CAU gene were identified. As a result of gene duplication, a total of 85 genes, including 47 protein-coding genes, 34 tRNA genes, and 4 rRNA genes were identified in the ginseng mitochondrial genome. Nucleotide composition analysis showed a slightly higher A + T bias, accounting for 54.9% (A, 27.1%; T, 27.8%; G, 22.5%; C, 22.6%). Phylogenetic analysis based on 18 protein-coding genes in the mitochondrial genome revealed that *P. ginseng* was closely related to *Daucus carota* in the Apiales (Figure 1). This complete mitochondrial genome sequence and genetic data will provide essential information for further studies related to the important medicinal plant *P. ginseng*.

**Figure 1.**
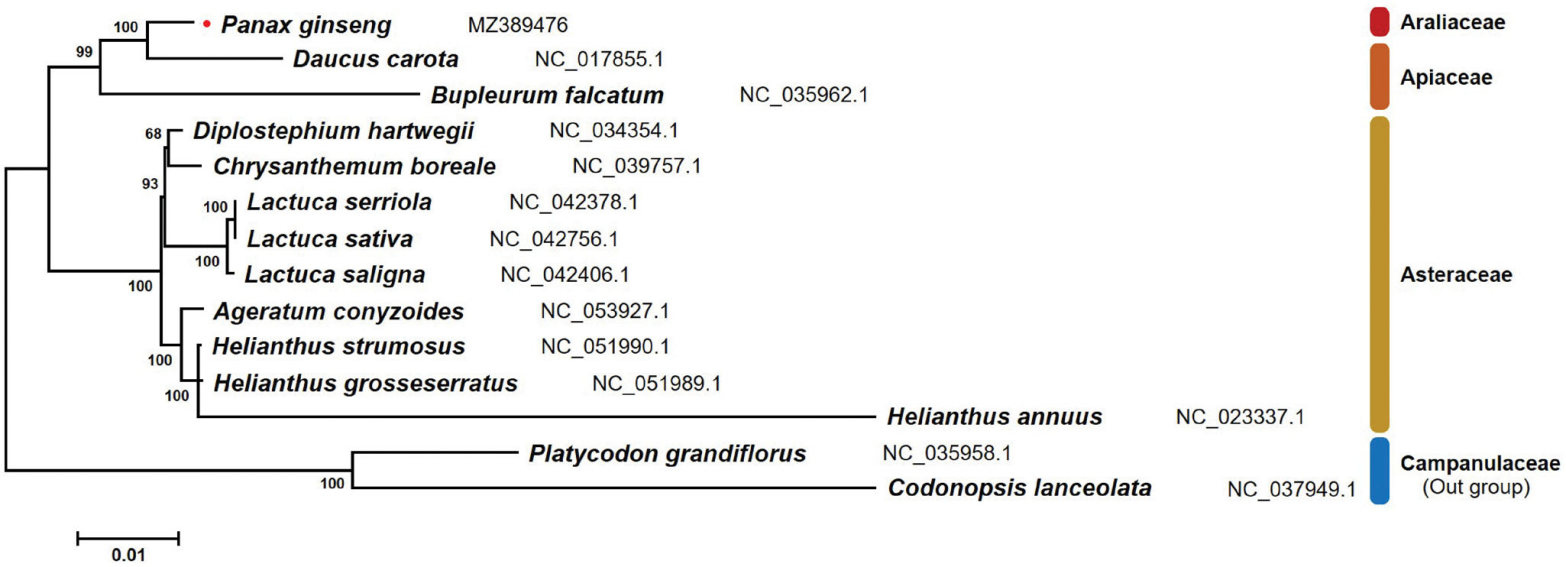
Phylogenetic relationship among 14 species based on 18 orthologous protein-coding genes in the mitochondrial genome. The numbers above the branches present the bootstrap value. The labels next to the scientific name of each species indicates the GenBank accession number deposited in NCBI database.

## Data Availability

The raw sequencing reads were deposited in the NCBI database under the BioProject accession number PRJNA737312 (https://www.ncbi.nlm.nih.gov/bioproject/PRJNA737312; SRA accession number for ONT and Illumina reads are SRX11135881 and SRX11135882, respectively). The mitochondrial genome sequence of *P. ginseng* landrace ‘Jakyung’ can be found in the GenBank data libraries under accession number MZ389476.
